# Sleep and Stroke—An Overlooked Bidirectional Influence: Why Should Sleep and Vascular Neurologists Work Closer?

**DOI:** 10.3390/jcm14207420

**Published:** 2025-10-21

**Authors:** Dario Bottignole, Carlotta Mutti, Liborio Parrino, Alessandro Pezzini

**Affiliations:** 1 Neurology Unit, Department of Medicine and Surgery, University of Parma, A. Gramsci Street 14, 43126 Parma, Italy; dario.bottignole@unipr.it (D.B.); liborio.parrino@unipr.it (L.P.); 2 Sleep Disorders Center, Department of General and Specialized Medicine, Parma University Hospital, A. Gramsci Street 14, 43126 Parma, Italy; carlotta.mutti88@gmail.com; 3 Mario Giovanni Terzano Interdepartmental Centre for Sleep Medicine, University of Parma, A. Gramsci Street 14, 43126 Parma, Italy; 4 Stroke Care Program, Department of Emergency, Parma University Hospital, A. Gramsci Street 14, 43126 Parma, Italy; 5 Department of Medicine and Surgery, University of Parma, A. Gramsci Street 14, 43126 Parma, Italy

**Keywords:** sleep medicine, stroke, sleep research

## Abstract

Since the earliest investigations into the impact of sleep-related breathing disorders on cardiovascular risk, the association between sleep–wake disorders and major cerebrovascular events has been increasingly and robustly established. Recent international joint statements—endorsed by leading scientific societies (e.g., American Heart Association, American Stroke Association, European Academy of Neurology, European Stroke Organization, European Sleep Research Society, and European Respiratory Society)—represent a milestone in stroke prevention and rehabilitation by formally recognizing sleep disorders as both risk factors for ischemic stroke and determinants of poor short- and long-term outcomes. Nevertheless, despite these strong epidemiological and mechanistic associations, the therapeutic evidence supporting sleep–wake interventions (e.g., positive airway pressure therapy, GABA-receptor agonists, melatonin) for stroke prevention remains limited and requires further validation through well-designed clinical trials. In this perspective article, we review recent advances in understanding the bidirectional relationship between sleep disorders and stroke, discuss the proposed pathophysiological mechanisms underpinning this complex interplay—with particular emphasis on arousal-related activation of the autonomic nervous system—and provide a critical appraisal of current research directions and future perspectives. Finally, we underscore the need for closer collaboration between sleep and stroke specialists to bridge existing knowledge gaps and optimize patient care.

## 1. Introduction

In 2015, the American Heart Association (AHA) and the American Stroke Association (ASA) first formally recognized sleep disorders (SDs) as significant risk factors for major cerebrovascular events [1], a position further reinforced in 2021 [2]. Around the same period, the American Academy of Sleep Medicine (AASM) also highlighted the close and bidirectional relationship between sleep and stroke [3]. Simultaneously, in 2020, a joint statement by leading European societies—the European Academy of Neurology (EAN), the European Respiratory Society (ERS), the European Stroke Organization (ESO), and the European Sleep Research Society (ESRS)—underscored the crucial role of SDs in stroke onset and prognosis [4]. More recently, both AHA and EAN publications have emphasized the broader pivotal contribution of sleep to overall brain health [5,6].

Both American and European publications revolve around the paramount evidence of an increased cardiovascular risk related to sleep-related breathing disorders (SBDs), mainly obstructive sleep apnea (OSA), which subsequently fostered the wider approach involving the other SWDs as well. However, further evidence has been limited by relevant methodological issues (e.g., frequent use of heterogeneous and unstandardized screening and diagnostic tools, restricted use of objective sleep evaluations, limited sample sizes, scarcity of data directly collected within a specific stroke population, incomplete control of potential confounding or modifying variables, etc.). Recent studies have begun to address these gaps while proposing several underlying pathophysiological mechanisms (intermittent hypoxia, reoxygenation damage, autonomic dysfunctions, alteration in cerebral blood flow dynamics, neuroinflammation, glymphatic system disruption) [7,8,9,10,11,12].

In this paper, we aim to examine literature from the last five years to build upon the 2020 European statement (Table 1 resumes all the described observations) and eventually encourage closer collaboration between vascular and sleep neurologists to overcome current knowledge gaps.

## 2. Review of Recent Evidence

### 2.1. Sleep-Related Breathing Disorders

According to the current version of the International Classification of Sleep Disorders (ICSD-3rd-TR), this category includes Sleep Apneas (SA), Central Hypoventilation Syndromes (CHS), Obesity Hypoventilation Syndrome (OHS), Sleep-related Hypoxemia Disorder (SHD), sleep-related hypoventilation due to other medical conditions, and isolated symptoms produced by breathing during sleep (e.g., snoring and catathrenia). However, from a cerebrovascular point of view, scientific literature revolved around AS (both obstructive and central) and OHS.

#### 2.1.1. Obstructive Sleep Apnea

OSA is the commonest sleep-related breathing disorder, affecting up to 1 billion people worldwide, with strong male predominance [13]. It is characterized by recurrent upper airway collapse during sleep, resulting in intermittent hypoxia, sleep fragmentation, and increased cardiovascular stress is an independent risk factor for stroke and all-cause mortality, with risk increasing progressively in relation to disease severity, regardless of other comorbidities, including hypertension. According to the International Classification for Sleep Disorders [14], OSA’s severity is graded by severity using the Apnea Hypopnea Index (AHI), which counts the number of respiratory events (apnea, hypopnea, and respiratory-related arousal efforts) per hour of sleep. The risk of stroke among the most severe OSA phenotype is estimated to be three times higher than in controls [15]; therefore, the AHA/ASA 2021 guidelines for the secondary prevention of stroke recommend treatment with Continuous Positive Airway Pressure (CPAP) to improve measures of sleepiness, blood pressure control, sleep-related quality of life, and physical functioning [2]. The association between the two conditions is not trivial, as the prevalence of moderate-to-severe OSA in patients affected by stroke approaches 40% [16]. While the impact of OSA on cardiovascular health is well established [2,4], the therapeutic effect of nocturnal ventilation on cardiovascular disease (CVD) risk remains uncertain due to conflicting evidence. Recent studies (both randomized controlled trials—RCTs—and observational ones) did not help to clear uncertainty, reporting contradictory outcomes: while some found no significant reduction in major cardio-/cerebro-vascular events (MACEs) regardless of treatment adherence [17,18,19,20,21], others displayed a significant reduction, particularly of non-fatal strokes, in adherent patients (≥4 h/night) [20,22,23]. Notably, seminal data support an increased risk of MACEs after ventilatory treatment termination [24].

These divergent results must be interpreted cautiously due to several limitations, including heterogeneous study designs, variable treatment adherence, selection bias from excluding severe cases, disparate sample sizes and follow-up periods, and underrepresentation of women and the elderly. Adherence to CPAP therapy is critical for accurately assessing its health benefits, as limited use of the device (<4 h per night) may leave patients vulnerable to pronounced autonomic disturbances during the latter half of the night, when REM sleep is most prevalent. Indeed, sleep is far from a uniform brain state, and it dynamically shifts from NREM to REM sleep stages, the latter dominated by strong sympathetic predominance [25]. The generalizability of RCTs is also limited, as patients enrolled in trials are usually strongly selected (i.e., less obese, older, with higher mean AHI, a higher prevalence of CVDs, and a lower incidence of non-cardiovascular comorbidities compared to common OSA patients) [26].

Two aspects of CPAP therapy warrant discussion. First, its role in cardiovascular prevention may differ between patients with and without overt CVD, showing a greater protective effect before MACEs occur and reduced benefit once cardiovascular damage is established [19,23]. Second, specific subgroups of OSA patients may benefit more from CPAP therapy than the general OSA population. In recent years, OSA patients have been grouped into “phenotypes” based on clinically relevant features (e.g., symptoms, therapy response, outcomes, quality of life) to guide personalized treatment: current classifications follow either a clinical approach (excessive daytime sleepiness, cardiovascular comorbidities, insomnia features) or a pathophysiological approach (upper airway collapsibility, arousal threshold, loop gain, muscle responsiveness) [15]. Although clinically relevant, these distinctions into phenotypes have never been considered in clinical trials, where OSA patients have instead been grouped into a single category, also because current diagnostic criteria for OSA do not account for phenotypic differences. Other strategies, including machine learning to integrate multiple predictors, are under investigation [27]. A fundamental limitation in previous OSA research is the over-reliance on the AHI, a metric that poorly captures the complexity of the disorder and demonstrates weak and inconsistent associations with cardiovascular disease risk [28]. The identification of severity markers beyond the AHI is currently a central focus of research in the field of OSA [28]. For instance, patients with a high baseline hypoxic burden (HB) [29], a high pulse rate response to respiratory events (∆HR) [30], or a shorter mean respiratory event duration [31] appear to gain a greater protective cardiovascular benefit from PAP therapy. The hypoxic burden is particularly relevant as it better reflects intermittent hypoxia’s impact on cerebral vascular dynamics and neuronal oxidative stress, thus correlating with cerebrovascular health [28,32,33].

These alternative metrics offer a more physiologically meaningful assessment of sleep-disordered breathing and its cardiovascular impact. Integrating such markers into clinical guidelines and trial frameworks could improve patient selection, enhance outcome interpretation and personalized risk stratification, and facilitate the translation of precision approaches into routine OSA care.

Reflecting on summarizing strategies, meta-analyses on the cardiovascular benefits of PAP treatment for OSA have also yielded conflicting results. An individual participant data (IPD) meta-analysis of 3 RCTs (4186 patients) showed that good PAP therapy adherence (≥4 h/night) had a beneficial effect in the secondary prevention of MACEs (HR 0.69 [95% CI: 0.52–0.92]) [34]. IPD are considered the gold standard for evidence synthesis, as they allow collecting and re-analyzing raw, individual-level data from each study included in the literature, rather than relying on published aggregate results, yielding detailed and consistent analyses across studies, with better control for confounders. These benefits are particularly important for a pathology such as OSA, which is deeply embedded with numerous concomitant health conditions. A more recent meta-analysis of 15 studies (9419 patients) demonstrated that PAP therapy significantly reduced the risk of MACEs and cardiovascular mortality in OSA patients compared to untreated individuals, for both primary and secondary prevention (MACE risk: RR 0.69 [95% CI: 0.54–0.89]; CV mortality risk: RR 0.53 [95% CI: 0.31–0.91]) [35]. In a meta-analysis of 30 records (117,615 participants), PAP therapy was significantly associated with a reduction in all-cause mortality (HR 0.63 [95% CI: 0.56–0.72]) and cardiovascular mortality (HR 0.45 [0.29–0.72]) [13].

Despite the contrasting data and current limitations aforementioned, most of them display a beneficial effect of CPAP therapy on individual cardio- and cerebro-vascular risk, particularly when it is conducted with good adherence and promptly started before the presence of an overt CVD. Thus, until further studies deliver more conclusive results and considering its overall safety, CPAP treatment shall be started as soon as possible, and it should be included in a multidimensional approach to cardiovascular risk factors control, comprising weight loss, anti-hypertensive medications, lipid-lowering drugs, and increased physical activity.

Lastly, a growing interest in the application of glucagon-like peptide-1 (GLP-1) receptor agonists in OSA patients is driving current research. Indeed, GLP-1 agonists displayed an unexpected effectiveness in treating morbid obesity and its comorbidities [36], and seminal data showed a reduction in OSA symptoms burden and disease severity (expressed as AHI value) associated with GLP-1 receptor agonists [37,38,39]. Current perspectives depict the weight loss induced by GLP-1 receptor agonists as the central mechanism underlying their beneficial role in OSA patients, also considering previous studies supporting the therapeutic effect of weight loss itself in obese OSA patients through reduction in AHI and systemic inflammation, but other potential mechanisms are under investigation (e.g., direct regulation of hypothalamic functions, reduction in sympathetic activity, independent reduction in systemic inflammation) [37,39]. Emerging data also depict a significant reduction in CVD risk, although further studies are mandatory [40].

Because impaired pharyngeal muscle activity during sleep plays a key role in the collapse of the upper airway in OSA, medications with noradrenergic and antimuscarinic properties (i.e., atomoxetine combined with oxybutynin), which directly affect pharyngeal muscle tone, are being studied worldwide for their potential to reduce OSA severity, with promising results [41]. However, despite their therapeutic potential, their cardiovascular effects will likely require careful monitoring.

#### 2.1.2. Central Sleep Apnea

Central Sleep Apnea (CSA) is a sleep-breathing disorder characterized by repeated episodes of apneas/hypopneas during sleep with no concomitant respiratory effort. A peculiar type of CSA is the Cheyne-Stokes respiration (CSB), also known as “periodic breathing”, described by a typical ventilatory pattern with at least a series of 3 consecutive CSA/CSH separated by periods of crescendo–decrescendo breathing with a cycle length from the onset of the apnea to the onset of the next of ≥40 s [42,43].

The only way to differentiate OSA and CSA is through a polysomnography, witnessing the absence of a respiratory effort during central events [43]. CSA is caused by a combination of increased loop gain (and subsequent overreaction of the respiratory system to chemical stimuli), reduced apnea threshold (with a higher risk of breathing cessation due to reduction in blood CO_2_ levels), and a narrower systemic CO_2_ reserve [44]. Such an unstable system leads to a direct alteration of neural respiratory drive, with subsequent apnoic/hypopnoic events generated by an abnormal activity of this specific central pattern regulator. Any medical condition potentially harming one of these three elements may lead to CSA: chronic heart failure, pulmonary hypertension, chronic kidney disease, stroke, medications (e.g., opioids, sodium oxybate, baclofen, valproic acid, ticagrelor), and even after initiation of non-invasive ventilatory treatments (so-called TE-CSA) due to a temporary alteration of CO_2_ balance [44]. However, a primary CSA syndrome is possible in patients without any of these predisposing conditions. Treatment of CSA is less standardized and more complex than OSA management, mainly because of its heterogeneous pathophysiology and the potential presence of other underlying medical conditions [45]. The AASM supports, with specific limitations, the following treatment options: positive airway pressure therapy, agents that modulate ventilatory control mechanisms (e.g., supplemental oxygen and acetazolamide), and implanted devices that stimulate the phrenic nerve [46]. A deeper analysis of each of these options goes beyond the purpose of this paper, but a crucial concept in CSA management that shall be stressed is the proper control of any underlying medical condition that could support CSA pathophysiology [45]. Notably, CSA can also exist as a defensive adaptive response to heart failure, and effectively treating CSA of adaptive servo-ventilation can cause more harm than benefit when compared to optimal medical therapy [47].

Regarding correlations with stroke, although previous reports suggested an increased risk of CSA after ischemic strokes and transient ischemic attacks, with variable incidence percentages [48,49], more recent evidence supports a lower prevalence of this disorder among stroke patients [50,51,52]. Several pitfalls could explain such an uncertainty: first, all these studies recruited large samples of patients with highly variable stroke sites and different recruitment time points, increasing results’ heterogeneity and subsequent comparability; second, the vast majority of studies about this specific topic include mainly patients with mild cerebrovascular events (i.e., NIHSS score < 6), leading to a selection bias through the exclusion of most severe ones; third, although relevant potential confounding factors have been effectively controlled, several others have been neglected, such as the use of medications that may alter the severity of nocturnal respiratory events. In general, there is a consensus that within 5 days after stroke, patients experience a higher risk of developing CSA, which progressively decreases in subsequent weeks/months, and then declines in prevalence and severity as the post-stroke period evolves, eventually becoming negligible later after [53].

Less is known about the significance of this early-onset respiratory pattern after stroke, and important clarification is expected from SleepSMART trials, which will evaluate whether CPAP initiation shortly after stroke or TIA reduces recurrent cerebrovascular events and all-cause mortality during 6 months of follow-up (Sleep SMART, ClinicalTrails.gov ID NCT03812653).

#### 2.1.3. Obesity Hypoventilation Syndrome

OHS is a condition in which severely overweight individuals fail to breathe adequately during sleep and wakefulness, leading to chronic low oxygen levels (hypoxemia) and elevated carbon dioxide levels (hypercapnia) in the blood. OHS has raised increasing concerns in recent years due to the global obesity pandemic [54,55]. From a pathophysiological perspective, a combination of impaired respiratory mechanics due to fat accumulation, leptin resistance, blunted respiratory drive, and limited lung expansion sustains the condition, which commonly overlaps with OSA, which furtherly worsens the hypoxic/hypercapnic events [54,56]. Compared to OSA, OHS is associated with poor prognosis and early mortality, which triggers the occurrence and worsening of cardiovascular and metabolic comorbidities and is associated with a higher risk of hospitalizations due to hypercapnic respiratory failure [54].

Previous studies have recognized a detrimental role of OHS on the autonomic nervous system (evaluated through monitoring of the HRV) [57] and associated it with chronic hypertension [58,59], but recent evidence showed a connection between nocturnal hypoxia and significant cognitive impairment through stimulation of local inflammatory responses, although it is still uncertain which is the precise underlying mechanism [60,61]. Unfortunately, most of the evidence about nocturnal hypoxia involves OSA patients, and not specifically OHS, raising potential concerns about the transportation of knowledge between these two distinct populations.

From a cerebrovascular perspective, previous evidence showed a significant increase in cardiovascular comorbidities among this population, but this evidence was restricted to major cardiac conditions (unstable angina, right ventricular failure, heart failure, and left atrial enlargement) and other cardiovascular risk factors (systemic hypertension and diabetes mellitus mainly), without a clear position on cerebrovascular impact, neither stated by the European scientific organizations [4,56,62].

Interestingly, although a common consensus about the proper therapeutic strategy for these patients is missing, recent evidence showed a better responsiveness of OHS than OSA patients to PAP treatment, with a stronger significant improvement of survival rates in the former group [63].

Considering the increasing incidence of obesity, particularly among young populations, we warrant the need for further studies specifically assessing the potential correlations between OHS and stroke, to better predict their individual cerebrovascular risk and improve prevention strategies among such a frail population.

### 2.2. Insomnia Disorder

Together with OSA, insomnia is among the most frequent sleep disorders both in the general population and in stroke patients, affecting up to 50% of stroke survivors [64,65,66]. It is crucial to distinguish insomnia disorder, as defined by the ICSD-3rd-TR [14], from the broader concept of insomnia symptoms. The former denotes a clinical diagnosis—classified as chronic, short-term, or other—based on symptom frequency (≥3 nights/week) and duration (≥3 months). The latter refers to subjective sleep-related complaints that may occur with comorbidities and do not necessarily meet diagnostic thresholds. Furthermore, below the umbrella term of insomnia, numerous distinct conditions are de facto included, such as insomnia with misperception of sleep, insomnia associated with objective short sleep duration or insomnia with/without hyperarousal. Unfortunately, these distinctions are commonly neglected in scientific literature.

Recent studies confirm the high prevalence of insomnia among stroke patients, ranging from 20% in those with no prior history to 40% in those with a previous diagnosis [66,67]. Post-stroke insomnia is associated with mood disturbances, low education level, female gender, and stroke severity [67,68], and recent studies linked it to more severe ischemic stroke, worse functional prognosis, and an increased risk of stroke recurrence and death [69,70,71,72]. Furthermore, post-stroke insomnia is also closely associated with mood disorders [73,74], potentially further harming rehabilitation and functional outcomes through the detrimental effects of depression itself [75,76].

Insomnia symptoms, including reduced total sleep duration (OR 0.53 [95% CI 1.14–2.04]), prolonged sleep onset latency (OR 1.50 [95% CI 1.14–1.68]), impaired subjective sleep quality (OR 1.39 [95% CI 1.18–1.75]), and more than one nocturnal awakening (OR 1.44 [95% CI 1.18–1.75]), are associated with an increased risk of severe stroke and poorer functional outcomes [71]. This is further supported by a meta-analysis showing that non-restorative sleep (HR 1.16 [95% CI 1.07–1.24]), sleep onset insomnia (HR 1.22 [95% CI 1.06–1.40]), and sleep-maintaining insomnia (HR 1.14 [95% CI 1.02–1.27]) are correlated with a significant risk of a first-ever cardiovascular event [66]. Consistent with this, another meta-analysis found that both sleep onset and sleep maintenance insomnia increased the risk of atrial fibrillation (OR 1.3 [95% CI 1.26–1.35]), hypertension (OR 1.11 [95% CI 1.07–1.16]), and large vessel occlusion strokes (OR 1.14 [95% CI 1.05–1.24]), and a subsequent umbrella review of 25 meta-analysis confirmed this evidence [74,77]. While there is some evidence suggesting a higher incidence of large vessel occlusion strokes in insomniac patients [70,77], no correlation with vascular territory has been established yet [72,78].

Impaired sympathetic nervous activity and disrupted inflammatory pathways may connect insomnia and ischemic stroke under a pathophysiological perspective, as evidenced by significantly higher inflammatory factors (e.g., C-reactive protein, white blood cell count, neutrophil count) and oxidative stress biomarkers (e.g., HIF-1α, MDA) in insomniac patients [70]. A systemic pro-inflammatory environment, an increased oxidative burden, and disruptions of the sympathetic activity related to sleep fragmentation and hyperarousal are also found in patients who develop post-stroke insomnia, potentially explaining the worse outcomes and higher recurrence risk these patients experience [69,70,71,72]. Recent studies also explored the potential involvement of the intestinal microbiota in post-stroke insomnia pathogenesis, possibly due to an increase in systemic inflammation via local triggering of immune response and impaired local production of neurotransmitters (which may also affect hypothalamic–pituitary–adrenal (HPA) axis and subsequently hamper the cortisol signaling, directly related to sleep–wake cycles and hyperarousal conditions) [79]. However, this evidence is still weak, and further studies involving larger samples are mandatory.

Given the potential role of insomnia in terms of cardiovascular risk, it is advisable to screen the condition among higher risk cohorts through uniform diagnostic criteria, considering differences in phenotypes and ideally combining subjective/self-reported with objective data (e.g., actigraphic recording, polysomnography).

#### Insomnia Treatment and Cardiovascular Risk

Current European guidelines for insomnia treatment recommend the non-pharmacological approach of Cognitive Behavioral Therapy for Insomnia (CBT-I) as a first-line approach for chronic insomnia, potentially associated with a pharmacological approach including benzodiazepines (BDZ), benzodiazepine receptor agonists, anti-orexinergic medications, and low-dose antidepressants for short-term treatment of insomnia. Prolonged-release melatonin is suggested for patients over 55 years old [80]. Other therapies (e.g., antihistaminergic drugs, antipsychotics, fast-release melatonin, ramelteon, and phytotherapeutics) lack sufficient evidence or are still limited to research settings.

Concerns persist regarding benzodiazepine (BDZ) use in stroke patients, as this class of drugs frequently hinders rehabilitation processes, particularly the regaining of walking independence [81,82,83,84]. A retrospective study of 290 post-stroke patients found that BDZs were mostly used for a short time (i.e., <7 days) among posterior circulation stroke patients, probably due to a direct involvement of structures related to sleep neurobiology, while patients with an anterior circulation ischemic stroke had a higher incidence of long-term (i.e., >3 months) use [84]. The study also noted a significantly higher prescription rate of Z-drugs (86%) compared to BDZs (14%), although Z-drugs could also negatively affect rehabilitation and functional outcomes [83].

Notably, in a Mendelian randomization analysis of a propensity score matched insomnia patient cohort from the UK Biobank (2544 patients), BDZ prescription has been correlated with an increased risk of coronary heart disease, heart failure, and cardiovascular death (but not stroke), while no association between Z-drug prescription and CVD risk was reported, suggesting a safer cardiovascular profile [85]. These conclusions should be taken cautiously, because of the several limitations that affected this study: it was not specified the precise BDZ prescribed, treatment duration, individual prescription duration, the severity of insomnia, and the number of potential covariates controlled during the analysis was relatively small. Furthermore, the authors collected the information from the UK Biobank cohort, which does not necessarily reveal causal associations; therefore, ad hoc studies covering this fundamental topic are warranted before writing any kind of hazardous conclusions.

In animal models, zolpidem has demonstrated promising effects regarding post-stroke insomnia, including sleep induction, reduced sleep fragmentation, and increased sleep depth. These effects might be particularly important considering the role of sleep on synaptic reorganization after brain injury and on neuroplasticity in general, which in turn may help to limit infarct extension and promote functional recovery [86]. Additionally, zolpidem has been shown to suppress cortical spreading depolarization, thereby limiting neuronal injury during reperfusion [87]. Similar benefits have also been observed in multidrug therapies with eszopiclone (mostly in association with Chinese compounds), which was shown to significantly improve post-stroke patients’ sleep quality in a network meta-analysis [88]. Accordingly, the most significant result can be obtained with the combination of eszopiclone with Sweet Dream Oral Liquid (a traditional Chinese herbal medicine).

In summary, although the evidence is still not conclusive, the potential hindrance of rehabilitation processes and the subsequent negative impact on regaining functional independence, as well as the reported possible increase in cardiovascular events risk, support current major concerns in the pharmacological treatment of post-stroke insomnia.

No studies since 2020 have evaluated melatonin as a hypnotic medication for stroke patients, but several publications have explored its potential neuroprotective role in both pre- and post-ischemic stroke models. Melatonin’s properties, including anti-oxidative effects, inflammation downregulation, mitochondrial activity promotion, and autophagy modulation, may protect neurons from ischemic insult, foster neuro-regeneration, and limit infarct volume [89,90,91,92]. These findings, however, are mostly based on animal model studies, as no human trials have been conducted to date.

Significant recent data have emerged on the effectiveness of digital CBT-I for treating post-stroke insomnia. Studies have demonstrated significant improvements in insomnia severity (ISI score), sleep quality (PSQI), depressive symptoms (PHQ-9), and anxiety (GAD-7) [93,94]. Although limited by factors such as missing OSA data and variable post-stroke enrollment times, these findings are promising and open to future potential effective and safer interventions in such a vulnerable population.

We advise that further studies should not only consider the potential neglected impact of other hypnotic medications, rather than BDZ and Z-drugs, but they should also address the possible role of other non-pharmacological strategies currently arising, such as transcranial magnetic and electric stimulation approaches, which are driving most of contemporary neuroscientific research in both sleep health and neurological disorders.

### 2.3. Restless Legs Syndrome and Periodic Limb Movements Disorder

Restless legs syndrome (RLS) is a sensorimotor disorder characterized by an uncontrollable urge to move the legs, usually accompanied by uncomfortable sensations temporarily relieved by voluntary movement [95]. Affecting up to 10% of the European population, its pathophysiology is linked to both brain iron deficiency and dopamine dysregulation, which are the main targets of therapeutic management [95]. Up to 85% of RLS patients also experience Periodic Limb Movements (PLM) during sleep [96]. When these movements disrupt sleep, they are classified as a disorder called Periodic Limb Movements Disorder (PLMD), which can occur independently of RLS [97]. RLS is among the most severe and drug-resistant causes of initial insomnia and frequently represents a challenge for sleep experts. Both RLS and PLMD conditions are associated with sympathetic hyperactivity, hypothalamic–pituitary–adrenal axis activation, and increased pro-inflammatory cytokines, and have been identified as potential risk factors for cardiovascular diseases, including hypertension and stroke [4,98].

#### 2.3.1. Restless Legs Syndrome

Evidence for a connection between RLS and stroke has not significantly grown since early international statements. The most consistent evidence suggests that both ischemic and hemorrhagic strokes can be pathogenic factors for a secondary “post-stroke RLS”. The prevalence of this complication varies across populations, from 2.3% to 15.1% in Asian populations and 5% to 13% in North American and European ones [99]. Patients with post-stroke RLS typically develop symptoms within two days of stroke onset; they have significantly higher NIHSS scores upon admission and one week after the event, along with a greater burden of depressive symptoms and lower quality of life [100,101]. The potential impact on independence and functional outcome at follow-up is still uncertain due to contrasting results [75,99,100,101]. Symptoms are usually unilateral, affecting the stroke-affected limb, and they often improve over several days to months, even without medication, although they are generally well managed with common dopaminergic and non-dopaminergic drugs (e.g., anticonvulsants, benzodiazepines, and opiates) [99,102,103]. Less is known regarding iron supplementation, which represents one of the key treatments for idiopathic RLS instead.

According to observational studies, stroke topography is frequently associated with the development of RLS, with subcortical lesions being the most common, particularly in the basal ganglia, corona radiata, internal capsule, thalamus, brainstem (pons), and cerebellum [99,100,102,103]. However, some Authors question the role of lesion location as a critical pathogenic factor, given the lack of significant differences between patients who develop RLS and those who do not, despite having vascular lesions in the same brain regions [99,100,103]. Pontine strokes may cause pathological recruitment of propriospinal or segmental spinal reflexes, while a functional disturbance of the sensorimotor network may lead to RLS symptoms regardless of the stroke location [99,101,103]. Other factors, such as significantly lower vitamin D levels, have also been identified as independent predictors for post-stroke RLS, pointing to a potential role of inflammation in its pathogenesis [104,105].

RLS is also frequently associated with uncontrolled resistant hypertension, particularly in young and female patients [106]. In post-stroke RLS patients, studies show a significantly higher systolic and diastolic blood pressure (both mean 24 h and nocturnal) compared to non-RLS patients [101]. Furthermore, obesity is an emerging risk factor, with a BMI >30 kg/m^2^ being associated with an increased risk of post-stroke RLS, especially in males [107]. Furthermore, RLS patients without a history of cerebrovascular disease have shown a significantly higher mean and maximum carotid intima-media thickness (IMT) and increased resistive index in both common and internal carotid arteries [108,109]. Additionally, RLS patients have demonstrated significantly higher mean and maximal middle cerebral artery velocities, suggesting an increased risk of major cerebrovascular events and cognitive decline [108]. Post-mortem studies further support these hypotheses by revealing a higher burden of microvascular disease in RLS patients compared to healthy controls [110].

A five-year prospective study of 24,199 patients with idiopathic Restless Legs Syndrome (iRLS) and no prior cardiovascular history found that while RLS itself is associated with an increased risk of major adverse cardiac events (MACEs), treatment significantly mitigates this danger. Specifically, the treated group showed a 13% net reduction in cardiovascular disease (CVD) risk (HR 1.26 [95% CI 1.20–1.30]) compared to the untreated group (HR 1.53 [95% CI 1.42–1.65]) [111]. This protective effect was observed across diverse treatment types, including dopaminergic agents, alpha-2-delta ligands, benzodiazepines, and opioids. Since these medications have varied effects on sleep and the autonomic system, it is hypothesized that the reduced cardiovascular risk is primarily due to the restoration of adequate sleep duration, which is often severely disrupted in RLS patients [111].

In summary, although further evidence is warranted, a proper sleep history collection should be helpful to detect covert sleep disorders such as iRLS in patients with uncontrolled CVDs despite multiple preventive and therapeutic strategies, particularly in middle-aged women and patients with resistant hypertension. A few questions proposed by the International RLS Study Group could help any physician in detecting potential RLS patients: “Do you ever feel an uncomfortable urge to move your legs?”; “Are these feelings worse when you are sitting still or lying down?”; “Do the symptoms get better when you move around, such as walking or stretching?”; “Are the sensations worse in the evening or at night compared to the daytime?” [112].

#### 2.3.2. Periodic Limb Movements Disorder

Limited literature exists on the link between PLMD and cerebrovascular events, with many studies focusing on detecting PLMs after a stroke rather than considering the role of this syndrome as a risk factor. The identification of PLMs, unlike RLS, requires polysomnographic recording, as it cannot be diagnosed based solely on clinical presentation, and the condition can be frequently silent from a clinical point of view. Consequently, observational data regarding this condition in the post-stroke setting are significantly limited. A prospective observational study found a high prevalence of PLMs (around 50%) in post-stroke patients, though it was unclear if this condition existed before the stroke [113]. Another study reported an even higher prevalence (76%) and found a correlation between PLMs and sleep-related breathing disorders, with the severity of the latter being a strong predictor for PLMs [114]. This study also found a significant negative correlation between PLM detection and excellent functional outcome, although it was not replicated in another parallel multicenter study [75,114]. While the link between PLMs and cerebrovascular health remains uncertain, a recent study found a significant correlation between the severity of the PLM index and the load of white matter hyperintensities expressed as Fazekas score and age-related white matter changes, confirmed with a multivariate ordinal logistic regression model (PLM index: OR 2.8 [95% CI 1.2–6.8]; PLM arousal index: OR 5.9 [95% CI 1.5–23.8]) [115]. Once the significance of PLMD in relation to cardiovascular risk is established, it will be important to determine whether this risk arises primarily from sleep fragmentation associated with the disorder or from the autonomic and cardiovascular stress induced by repetitive involuntary movements. This distinction is clinically relevant, as commonly used treatments (i.e., alpha-2-ligands or clonazepam) primarily target sleep fragmentation by reducing arousals or suppressing limb movements.

### 2.4. Parasomnias

Insufficient evidence still exists regarding a potential correlation between parasomnias and individual cerebrovascular risk since the EAN/ERS/ESO/ESRS statement was published. Most studies have focused on REM sleep parasomnias, particularly REM Behavior Disorder, leaving the role of NREM sleep parasomnias largely unknown.

REM sleep Behavior Disorder (RBD) is characterized by dream-enacting behaviors due to the loss of muscle atonia during REM sleep and is considered part of the synucleinopathy spectrum [116]. A key feature of synucleinopathies is autonomic impairment: a recent prospective study confirmed that patients with idiopathic RBD (iRBD) also exhibit autonomic dysfunction, primarily in blood pressure regulation, showing a significantly reduced nocturnal dipping profile (*p* = 0.026 for systolic and *p* = 0.001 for diastolic blood pressure) and a higher frequency of non-dipping status (*p* = 0.01), which are related to an increased cardiovascular risk [117]. Furthermore, in a historical community-based study, it has reported an increased risk for ischemic (HR 1.93 [95% CI 1.07–3.46]) and hemorrhagic (HR 6.61 [95%2.27–19.27]) stroke for RBD patients [98,118]. A recent analysis based on genome-wide association studies found a significantly increased risk of cardiovascular diseases in genetically predicted RBD, though this was only relevant for myocardial infarction and heart failure, not stroke [119]. On the other hand, although some past case reports have suggested that brainstem strokes can trigger RBD, the anatomical and pathophysiological pathways supporting a potential “post-stroke RBD” are still uncertain [98,120,121].

Once again, the significant heterogeneity and limitations of current studies compromise the reliability of the aforementioned findings, although they support an increased CVD risk in this specific population, and further research is needed to explore the potential correlation between parasomnias and cerebrovascular disorders, as well as the possible beneficial effect of medications such as melatonin.

### 2.5. Central Disorders of Hypersomnolence

Excessive daytime sleepiness (EDS) is the clinical hallmark of Central Disorders of Hypersomnolence, which include primarily Narcolepsy type 1 (NT1), Narcolepsy type 2 (NT2), Idiopathic Hypersomnia (IH), and Klein-Levin Syndrome (KLS) [122]. The discovery of orexin deficiency as a key pathophysiological factor in NT1 significantly advanced the understanding of sleep–wake regulatory features and connections between this disorder and cardiovascular risk [123].

Real-world evidence on the correlation between central hypersomnia and cerebrovascular events remains limited. Previous studies have suggested a potential increased risk of major cerebrovascular events in patients with NT1 and NT2, with some authors proposing orexin deficiency as a possible molecular link between NT1 and increased cardiovascular risk [98,124,125]. A recent retrospective analysis of US adult narcoleptic patients found a significantly higher risk of ischemic stroke (HR 1.67 [95% CI 1.19–2.23]) compared to healthy controls, with similar findings for both NT1 and NT2 patients [126]. Another retrospective analysis obtained comparable results (adjusted HR for any stroke: 2.06 [95% CI 1.73–2.45]), also showing a slightly higher risk for NT1 patients compared to NT2 [127]. In contrast, a two-sample Mendelian randomization study found a significant association between narcolepsy and increased cardiovascular disease risk, but specifically not with ischemic strokes [128].

Despite the observed increase in cardiovascular risk among patients with NT2, the underlying mechanisms remain largely unexplored. A recent cross-sectional study from Japan involving 83 NT2 and 57 IH patients reported a higher prevalence of metabolic syndrome-related comorbidities—such as hypertension, diabetes, and hypercholesterolemia—among the NT2 group [129], suggesting that metabolic dysfunction may contribute to the elevated risk of MACEs.

The cardiovascular risk among hyper-somnolent patients might also be related to medications adopted to treat these conditions: modafinil, for instance (diphenylmethyl-sulfinyl-acetamide), is a psychostimulant drug used for the treatment of daytime sleepiness in narcolepsy and is known to increase heart rate and blood pressure, eliciting sympathomedullary activation. In contrast, solriamfetol, a wakefulness-promoting agent inhibiting dopamine and norepinephrine reuptake, seems safer with respect to cardiovascular parameters [130]. A consensus on the role of NT and other central disorders of hypersomnolence in cerebrovascular health is urgently needed, especially for patients with a long history of treatment, given the potential for addictive and detrimental effects on overall cardiovascular health from traditional therapies like methylphenidate and modafinil [131,132].

### 2.6. Circadian Rhythm Sleep–Wake Disorders and Abnormal Sleep Duration

#### 2.6.1. Circadian Rhythm Sleep–Wake Disorders

Circadian Rhythm Sleep–Wake Disorders (CRSWDs) occur when the body’s internal clock becomes misaligned with environmental cues, or “zeitgebers,” such as light and social schedules, leading to a disruption of sleep–wake cycles and hormonal stability [133]. Melatonin plays a crucial role in coordinating the coupling between all these inputs, representing the so-called “chronobiotic signal”: the reduction in light stimulation to the suprachiasmatic nucleus induces its synthesis and release, promoting sleep onset and subsequently modulating circadian phase, thus being protective against developing a CRSWD and representing a cardinal treatment strategy [133].

CRSWDs are categorized as either intrinsic (due to an internal clock issue) or extrinsic (due to environmental factors), with specific subtypes including delayed or advanced sleep phase disorders and shift-work sleep disorder [14,133]. In the pathophysiology of these disorders, the individual’s unique “chronotype” (i.e., preferred timing for sleep and other functions, typically categorized as a morning, evening, or intermediate type) is fundamental [134].

A growing body of evidence suggests a direct association between circadian biology disruption and cerebrovascular events [135,136]. The underlying mechanisms are complex and involve multiple pathways. Circadian fluctuations directly affect blood–brain barrier permeability and physiology [137,138], while clock genes play a fundamental role in cerebrovascular stability, regulating the local immune system and promoting a morning prothrombotic state [138]. Disrupted circadian rhythms also alter inflammatory mediators, both systemically and in the central nervous system, which are directly connected to modifications in the cerebral hemodynamics [10]. Based on animal models, inflammation may act as a mediator between CRSWDs and cerebrovascular health, with CRSWDs and abnormal cytokine expression associated with increased mortality and poorer recovery after an experimental cerebrovascular injury, particularly in females [139]. Although clock gene expression appears largely preserved after acute ischemic stroke [140], the role of melatonin as a prognostic marker remains uncertain. Some studies show no significant differences in melatonin after a cerebrovascular event [140], while others report a delayed dim-light melatonin onset (DLMO) in the acute phase, leading to delayed sleep–wake disorder [141]. When melatonin secretion is delayed in the evening, the individual’s biological night shifts later, leading to several physiological, psychological, and functional consequences. The circadian misalignment itself may increase the risk of cerebrovascular events, due to its connections with worse chronic hypertension control, impacting both cardiac and cerebrovascular health [142]. Interestingly, it has been hypothesized that physiological circadian rhythm may also directly influence the timing of stroke onset: the presence of exogenous factors such as orthostatic changes (and related autonomic modifications) and endogenous variations such as the fluctuations in blood pressure and a relative pro-thrombotic state, have been related to the highest incidence of stroke occurring in the morning [42]. Furthermore, the presence of CRSWDs increases the risk of physical frailty in middle-aged adults, negatively impacting post-stroke functional prognosis [143].

The Shift-Work Sleep Disorder warrants a particular focus nowadays: it affects more than 20% of workers and is a reason for growing concerns due to its detrimental effects on overall health and cardiovascular and cerebrovascular wellness [144,145]. It is caused by the chronic misalignment of the body’s circadian rhythm due to repetitive alterations in work schedules [145], and patients have a significantly increased risk of accumulating several cardiovascular risk factors (e.g., smoking, high body mass index, high body fat percentage, type 2 diabetes mellitus, hyperlipidemia, atrial fibrillation, coronary heart disease), being the frequency of late-night shifts and the total duration of shift working potential predictors of such a predisposition [146,147,148,149]. Furthermore, shift work appears to be associated with a poorer functional outcome in stroke patients three months after the event [147]. This evidence warrants a reflection among physicians, who should consider the role of modern work as a potential source of illness.

#### 2.6.2. Abnormal Sleep Duration

Recommended average sleep duration for healthy adults is 7–9 h per night [150]. While individual sleep needs vary and durations outside this range are not necessarily pathological until there are no daytime complaints, a clear quantitative consensus on what defines “pathologically” long or short sleep has not been reached yet, also due to significant geographical variations [151,152]. The AASM has emphasized the potential negative health effects of short sleep duration, a concern supported by recent evidence [150,151,153].

An increased risk of both ischemic and hemorrhagic stroke is associated with extreme sleep durations, specifically less than 5–6 h or more than 9–10 h per night [154,155,156,157,158,159,160,161,162,163,164,165,166,167,168]. This U-shaped relationship persists after a stroke, with extreme sleep durations linked to higher odds of post-stroke depression and poorer functional and psychological outcomes in survivors [169,170]. This association appears to be more significant in females and younger patients, as well as those with a poorer clinical status before the stroke [154,160,164,168,171]. Current evidence suggests chronic inflammation may be the link between extreme sleep durations and stroke risk, as indicated by elevated inflammatory biomarkers in these patients [172,173]. Chronic inflammation can both promote atherosclerosis and disrupt lipid metabolism, leading to increased carotid plaque burden, reduced arterial elasticity, and impaired blood pressure control [164,172,173,174].

Although the evidence regarding the role of CRSWDs on cerebrovascular health is still growing, the historical amount of evidence regarding the U-shape correlation of sleep duration with CVD risk supports their investigation in any preventive approach, although subjective perception could significantly differ from the objective duration, leading to interesting perspectives about the large population use of wearable devices as screening tools.

## 3. Pathophysiological Perspectives

Several pathophysiological factors have been proposed to explain the bidirectional interplay between sleep–wake disorders and stroke. A major cerebrovascular event may involve a specific site, leading to sleep disruptions such as hypersomnolence or circadian rhythm disorders through a selective impairment of neurological functions regulated by that affected site [98,175], but it may also lead to a whole network dysregulation, affecting more complex and widespread functions, such as sensory-motor integration and default-mode activity, and subsequent heterogeneous and unpredictable sleep–wake disorders such as post-stroke insomnia and post-stroke Restless Legs Syndrome [99,103,175]. Sleep-related Breathing Disorders, Sleep Apnea primarily, could be triggered or worsened after a stroke through several mechanisms: direct affection of critical areas regulating upper airway patency or breathing effort, neurotoxic effect exerted by excessive excitatory amino acids released after neuronal necrosis, abnormal forced postures due to physical limitations, hyperventilation-related hypocapnia due to an increased intracranial pressure, and subsequent abnormal respiration during sleep [175,176,177]. Notably, even without direct involvement, after a stroke event, an alteration of outputs from the pontomedullary respiratory pattern generator to the respiratory muscles leads to an increased incidence of Central Sleep Apnea [177].

On the other hand, the role of sleep disorders as cardiovascular risk factors revolves around two crucial elements: the autonomic nervous system and the immune-inflammatory system [173]. In the post-stroke context, sleep disturbances can profoundly disrupt autonomic homeostasis by impairing sympatho-vagal balance through several interrelated mechanisms, including paradoxical sympathetic hyperactivation during sleep, hypothalamic–pituitary–adrenal (HPA) axis dysregulation, increased arousal burden, and recurrent episodes of intermittent hypoxemia and hypercapnia—particularly in patients with coexisting sleep-disordered breathing [173,178,179,180]. Such an altered nocturnal sympatho-vagal balance is associated with an increased risk of hypertension, tachyarrhythmias (atrial fibrillation primarily), systemic endothelial dysfunctions, and coronary artery disease [173,178,179,180]. Sleep disorders are also associated with an increased systemic immune and inflammatory dysregulation, mainly due to hyperexpression of pro-inflammatory mediators and cytokines (e.g., IL-1β, IL-2, IL-6, TNF-α, IL-18, sTNF-R1, and sTNF-R2), and intermittent hypoxia-induced oxidative stress [60,173,178]. Such abnormal immune activation may also be observed within the brain, as evidenced by seminal reports of immunologic alterations in patients with sleep disorders [110] and a potential pre-stroke blood–brain barrier disruption, which alters the endothelial homeostasis, inducing a local pro-thrombotic state [60].

Although the strength of evidence is smaller, another remarkable dysregulation associated with sleep disorders regards the cerebrovascular reactivity, with a greater vascular contractile tension and an impaired flow-mediated vasodilatation, due to both a direct alteration of endothelial molecular homeostasis and an indirect inflammatory-mediated damage [60,173,178]. Similarly, patients affected with sleep disorders (mainly: SBDs, CRSWDs, extreme sleep durations) tend to present a metabolic imbalance (with higher rates of insulin resistance, obesity, hypercholesterolemia, and diabetes mellitus) and direct coagulation impairment [60,173,178].

**Figure 1:**
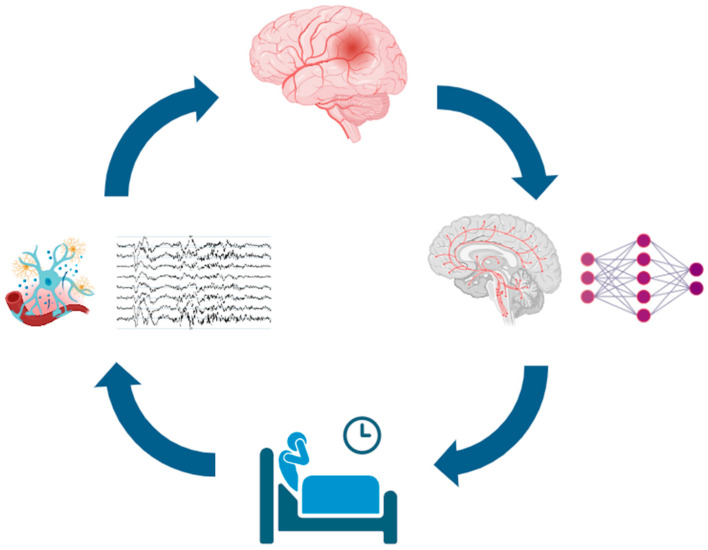
Schematic of the bidirectional relationship between sleep disturbances and acute cerebrovascular events. Sleep disturbances (bottom) may promote neuroinflammation and nocturnal cortical and autonomic arousals (left), increasing the risk of acute cerebrovascular events (top). These events can damage localized sleep-regulatory structures rather than broader neuronal networks (right), further impairing sleep and creating a self-reinforcing cycle. Icons from https://BioRender.com (free trial version, accessed on 11 October 2025), modified and adapted with https://www.canva.com/ (accessed on 11 October 2025), EEG traces were personally obtained from the First Author (DB) sleep recording.

In summary, current pathophysiological evidence indicates that neuroinflammation and autonomic dysregulation represent two key mediators between sleep disorders and cerebrovascular disease. Figure 1 illustrates the pathophysiological interplay between sleep disorders and acute cerebrovascular events.

### Nocturnal Arousals

Despite the complexity of the pathophysiological mechanisms and existing gaps in current knowledge, the pursuit of a unifying framework linking sleep and cerebrovascular health remains a compelling and evolving area of research, with each advancement shedding light on potential integrative pathways. A cardinal role in this framework is certainly played by nocturnal arousals, brief intrusions of wake-like brain activity coupled with changes in heart rate, respiration, and muscle activation during sleep [181]. These events might be disruptive enough to restore complete wakefulness, as well as shorter in time and magnitude, the so-called “microarousal”, which represents a physiological sleep feature [181]. According to the American Academy of Sleep Medicine, these events are scored whenever there is an abrupt shift in EEG frequency, including alpha, theta, and/or frequencies greater than 16 Hz, which lasts for 3–15 s [43]. Although this definition is focused on a cortical perspective, autonomic fluctuations are always tightly coupled to such changes [182], in a complex coupling system widespread through several cortical and subcortical sites and networks [181,183,184,185].

If we analyze sleep from a different perspective, if we move away from the conventional framework that segments sleep into 30 s epochs and defines arousals strictly as brief, rapid events, we can appreciate that arousals are physiological elements of the sleep micro-structure, and rather than being isolated events across the whole night, they tend to organize in physiological periodic sequences, defined as “Cyclical Alternating Pattern” (CAP), with a 2–60 s periodicity [186,187]. This periodic activity is traditionally recognized as a marker of sleep instability and resilience towards disruptions [182,185,187,188], and recent publications reinforced the correlation between CAP and sleep quality [189,190]. This pattern, rather than being composed of only rapid frequencies as in the AASM rules, includes both slow waves and fast rhythms, reflecting the natural oscillations of the cortical activity in the sleeping brain in a much more realistic fashion. The difference is significant, as the sleeping brain responds to potential intrusions—whether external or internal—not solely with brief fast arousals, but initially through slow-wave activity. This may manifest as isolated events, such as K-complexes, or as sequences with a periodic pattern. In other cases, the response involves fast EEG activity, depending on the intensity and duration of the stimulus as well as the sleep stage. Autonomic parameters fluctuations (e.g., heart rate, blood pressure, respiratory activity, cerebral blood flow dynamics, etc.) are also registered, coupled with CAP periodicities [182,185,187], and seminal evidence is emerging about its coupling with other physiological cerebral features, such as intracranial pressure oscillations [191,192]. Across the whole night, the human brain activity alternates during NREM sleep from cyclic periods (CAP sequence) to non-cyclic periods (non-CAP sequence). CAP is organized into cycles, each composed of an A phase (activatory) and a B phase (return to background EEG activities). CAP phase A might further be subdivided, according to their electrophysiological composition in subtype A1 (dominated by slow waves, generally associated with a weak autonomic and motor activation), A2 (a mixture of slow wave and fast frequencies, with intermediate vegetative activation) and A3 (characterized mostly by alpha, beta or faster EEG activities, usually associated with stronger autonomic and behavioral reactions) [185,186,187].

Taking into consideration the impact arousals have on nocturnal autonomic stability, any condition hampering sleep stability and increasing arousal frequencies or magnitude may exert a detrimental effect on the nocturnal autonomic balance, leading to paradoxical sympathetic activation and increased risk of cardiovascular consequences as explained above [178,193].

Notably, in past times has been demonstrated that CAP A phases, independently from phase A subtypes, are associated with a significant cardiovascular impact that peaks from 4 to 8 s after the onset of phase A, with greater impact on heart rate and blood pressure changes during A3 subtypes [194,195].

As a considerable amount of evidence has proved over decades, a significant number of sleep disorders are traditionally associated with alterations of arousal networks, witnessed by an objective impact on CAP parameters (mainly CAP time and CAP rate, and indexes of each A phase subtype, as displayed in Table 2) [182,187,196].

Hence, a disruption in the physiological arousal mechanism should never be observed just as representative of increased sleep fragmentation (and thus a reduced sleep quality) but rather be contextualized in the general framework of the cardiovascular and autonomic system, in a vertically integrated approach. Recent evidence showed a close coupling between cardiopulmonary pathophysiology and CAP, proven by an association between the main heart rate frequency bands and CAP/non-CAP sequencies (i.e., prevalence of low frequencies during CAP periods and high frequencies during non-CAP periods) [185]. If such evidence may support the potential use of Heart Rate Variability (HRV) as a marker of sleep quality, it reinforces the crucial hypothesis that behind a CAP alteration lies a series of autonomic alterations, together with cerebral hemodynamic modifications, that directly affect the individual cardiovascular risk.

Changes in nocturnal arousal dynamics are often overlooked during routine polysomnography studies, but they connect the cortical activity with individual cardio-cerebro-vascular health through the autonomic nervous system, and the correct assessment may help in improving cardiovascular risk stratification both in experimental and clinical settings. From this point of view, evaluating the magnitude of the autonomic response associated with the cortical arousal is fundamental. The HRV is still the most common autonomic marker used both in clinical and experimental situations to assess the wellness of the sympatho-vagal balance, correlating also with the individual cardiovascular risk [197], but several other techniques studying the autonomic nervous system might be used during nocturnal recordings (e.g., the pulse wave amplitude—PWA, the pulse transit time—PTT, the peripheral artery tonometry—PAT, the nocturnal short-term BP variability, etc.) [183,185,198,199,200]. Future research should further explore such pivotal coupling to better understand the role of nocturnal arousal and sleep fragmentation in determining individual cardiovascular risk and potentially integrate such an approach with cerebrovascular dynamic studies (e.g., transcranial Doppler), in order to also open perspectives of therapeutic approaches based on restoring sleep stability to reduce the cardio- and cerebro-vascular risk.

## 4. From Limitations to Future Directions

The bidirectional relationship between sleep disorders and cerebrovascular diseases is increasingly recognized, yet the current body of evidence is limited by methodological heterogeneity, underrepresentation of vulnerable subgroups, and a scarcity of stroke-specific investigations. While recent international consensus statements have emphasized the relevance of sleep in vascular health [3,4,5], substantial knowledge gaps remain that require a coordinated research agenda that shall integrate both Sleep Medicine and Vascular Neurology specialists’ needs.

A major limitation of the existing literature concerns the lack of standardization in diagnostic and screening tools for SDs: studies have frequently relied on self-reported questionnaires or heterogeneous methodologies, limiting reproducibility and cross-comparability. Objective and reproducible measures (e.g., polysomnography, wearable technologies, and validated biomarkers) should be more widely integrated into future protocols, including advanced analytic frameworks that consider further testing variables (e.g., hypoxic burden and nocturnal arousal dynamics) to refine cardiovascular and cerebrovascular risk stratification beyond traditional indices.

A remarkable gap in current knowledge about the interplay between sleep disorders and cerebrovascular events regards nocturnal epilepsies, and eminently the sleep hypermotor epilepsy (SHE). In past years, different studies approached the cardio-/cerebro-vascular risk of epileptic patients, reporting an increased risk of both ischemic and hemorrhagic events in this specific population [201,202], with variable risk ratios in different subtypes of disease [203]. Animal model studies have hypothesized a central role of uncontrolled hypertension underlying such an association, potentially due to an up-regulation of angiotensin II type 1 receptor and angiotensin-converting enzyme [204], but population-based studies have reported a significantly increased incidence of several CVD risk factors besides hypertension [202,203]. Furthermore, anti-seizure medications have been associated with an increased stroke risk, particularly phenytoin, sodium valproate, oxcarbamazepine, levetiracetam, phenobarbital, and carbamazepine [201,205]. Different potential mechanisms supporting this latter evidence have been proposed, but two are the leading hypotheses: the disruption of lipid metabolism due to a drug-related enzyme-inducing activity, and direct alterations of cardiac conduction through the influence of ion channels’ activity (particularly sodium channel blockers) [205]. Although several pieces of evidence have been collected regarding this topic, no studies have specifically focused on nocturnal epilepsies, leading to a significant knowledge gap which we encourage exploring in the near future through ad hoc studies, to better stratify the individual cerebrovascular risk of these patients who may experience an exponential risk increase due to the summatory influences of epilepsy and sleep disruption.

Most of the current evidence derives from cardiac populations, which limits its external validity for cerebrovascular cohorts. As depicted above, stroke survivors present unique pathophysiological features, including lesion-specific disruption of sleep–wake networks, altered cerebral blood flow regulation, and recovery processes dependent on sleep integrity. Dedicated prospective studies within acute and chronic stroke populations are therefore warranted. Such studies should evaluate not only the prevalence and prognosis of SDs, but also the efficacy and safety of therapeutic interventions still largely neglected, such as mandibular advancement devices, physical approaches, orexin-receptor antagonists, dopaminergic agents for restless legs syndrome, cognitive behavioral therapy for insomnia, and novel approaches including circadian rhythm modulation and neurostimulation.

Future investigations must also address heterogeneity across subgroups. Sex-specific and age-specific analyses remain insufficient, as well as subgroups stratification of stroke patients according to the event type (ischemic versus hemorrhagic) and the etiology (cardioembolic, large vessel atherosclerosis, small vessel occlusion, undetermined). Regarding this latter, a widely neglected topic that warrants further future research is the potential influence of specific therapeutic strategies for different strokes (e.g., endovascular treatment, intravenous thrombolysis, antiplatelet therapy) on sleep–wake health.

Finally, broader societal and lifestyle determinants urge more consideration: circadian disruption linked to shift work, digital exposure, and chronic sleep restriction represents a modern health challenge with significant cerebrovascular implications. Addressing these factors requires not only clinical vigilance but also coordinated public health strategies.

Progress in this field ultimately requires stronger collaboration between Sleep Medicine and Vascular Neurology specialists. Joint research registries, multicenter randomized trials, and harmonized diagnostic frameworks are essential to overcome current fragmentation. Embedding systematic sleep evaluations into stroke care pathways could improve acute management, rehabilitation, and long-term outcomes, while incorporating vascular risk assessment in sleep clinics may aid early identification of high-risk individuals.

## 5. Conclusions

Ten years have passed since the first statement from the AHA/ASA, and five since the European one. However, sleep disorders in stroke patients are still widely under-reported and under-diagnosed. To overcome such a gap, education and research are crucial: in both cases, a close collaboration to bridge the domains of sleep medicine and vascular neurology should be enhanced, not only to foster a mutual professional growth but mainly to play a cathartic role in better prevention and care of cerebrovascular and sleep–wake disorders. Prolific reciprocal co-operation is the key strategy to fill current knowledge gaps and, through progression, ensure patients multidimensional wellness.

## Figures and Tables

**Table 1:** Summarizing table reporting a concise synthesis of observations described above. Previous international statements refer to the 2020 EAN/ERS/ESO/ESRS and 2021 AHA/ASA publications [2,4].

Sleep Disorders		2020 International Statements	Current Knowledge
Obstructive Sleep Apnea	Stroke risk	Untreated severe OSA doubles the risk of incident stroke. Such risk appears especially relevant in young to middle-aged patients, without differences between men and women.	Untreated OSA is one of the most neglected risk factors, especially in young/middle-aged adults. Up to 50% of stroke patients may experience post-stroke OSA, potentially increasing the risk of secondary events.
	Treatment influence	Observational cohort studies suggest that CPAP treatment is associated with a reduced risk of stroke in patients with OSA, but the results are very variable. In meta-analyses, CPAP treatment is not associated with stroke risk reduction; however, patients adherent to CPAP therapy (>4 h per day) may benefit. There is insufficient evidence on treatment options other than CPAP.	Patients without an overt CVD may benefit more from PAP treatment, although data about secondary prevention are still somewhat uncertain. Good PAP adherence (≥4 h/night) is protective against CVDs. Improper PAP termination is associated with an increased risk of MACEs. GLP-1 receptor agonists may have a CV protective effect in OSA patients, although data are still scarce. OSA patients’ phenotypization may help to select specific subgroups with better prognosis.
Central Sleep Apnea	Stroke risk	Not enough data to express a detailed position.	Whether CSA impacts the individual risk of CVDs is still uncertain due to contrasting evidence. But several methodological biases hamper conclusions. During the post-stroke acute phase, patients experience a higher incidence of CSA, with seminal data reporting an increased risk of mortality due to stroke recurrence, but evidence is still limited.
Obesity Hypoventilation Syndrome	Stroke risk	Not included in international statements.	OHS was associated with a higher risk of chronic hypertension, diabetes mellitus, and major cardiac conditions. Evidence about the impact on stroke risk is still insufficient.
Insomnia disorder	Stroke risk	Insomnia slightly increases the risk for cardiovascular events, but the risk for stroke is uncertain. Prospective studies evaluating the association of insomnia with stroke risk are scarce, and all available studies are based on subjective measures to define insomnia, thus reducing the accuracy of exposure identification. There is a link between post-stroke insomnia, female gender, and depression	Insomnia is associated with an increased risk of stroke, particularly sleep onset and sleep maintenance insomnia. LVO stroke is the most frequent type, but no correlation with the affected vascular territory has been shown. Post-stroke insomnia affects up to 40% of patients and is correlated with more severe strokes, a higher risk of post-stroke depression, and worse functional outcomes.
	Treatment influence	Treatment of insomnia with BDZ/BDZRs is linked with an increased risk of cognitive dysfunction, dementia, and mortality, and possibly also stroke, especially in high doses and long-term use. This effect may also be related to an indication bias (patients who are in a worse general or neurological condition suffer more frequently from insomnia and may receive BDZ/BDZRs more frequently). There are no systematic data on the effect of insomnia treatment on stroke outcome; worsening of neurological deficits with hypnotics has been reported.	BDZ treatment hinders rehabilitation processes, particularly regaining walking independence. Large biobank analysis associated post-stroke BDZ treatment with an increased risk of CVDs (but not specifically stroke). Z-drugs displayed safer profiles on CV and rehabilitation outcomes, particularly for zolpidem and eszopiclone. No data regarding melatonin and DORAs are available. Preliminary data suggest a potential positive effect of repetitive TMS and digital CBT-I on sleep quality, insomnia symptoms, and depressive manifestations, although data are still limited.
Restless Legs Syndrome	Stroke risk	Current evidence does not suggest an increased risk of stroke in patients with RLS.	Preliminary data support an increased risk of stroke among untreated RLS patients. Uncontrolled hypertension, higher mean and maximum carotid intima-media thickness, increased resistive index in carotid arteries, and higher mean and maximal MCA velocities could be potential underlying mechanisms.
	Treatment influence	Based on the lack of evidence, no statement can be made.	A single prospective observational study reported a significant protective effect of almost all treatment options (dopaminergic agents, alpha-2-ligands, BDZ, and opiates) against CVDs.
Periodic Limb Movements Disorder	Stroke risk	Periodic limb movements in sleep may represent an independent risk factor for stroke.	Although some studies reported an increased incidence of PLMs after ischemic strokes and their correlation with worse functional outcomes, data regarding pre-stroke PLMs and their impact on stroke risk are still lacking.
	Treatment influence	Based on the lack of evidence, no statement can be made.	No further progress has been made.
Parasomnias	Stroke risk	Not included in international statements due to insufficient data.	RBD exerts a detrimental effect on blood pressure control, increasing the risk of CVDs. A historical community-based study reporting an increased risk of both ischemic and hemorrhagic stroke in RBD patients is the only reliable evidence so far.
Central Disorders of Hypersomnolence	Stroke risk	Not included in international statements due to insufficient data.	NT1 adult patients might experience an increased risk of stroke, although data are still limited. NT2 and IH patients seem to have a higher incidence of metabolic syndrome-related comorbidities, but not a clearly increased risk of MACEs.
Circadian Rhythm Sleep–Wake Disorders	Stroke risk	Not included in international statements.	Although several mechanisms have been proposed to explain the connection between CRSWDs and stroke risk, the only structured data available so far regards Shift-Work Sleep Disorders, which is associated with the accumulation of several CVDs and worse long-term functional outcomes in stroke patients.
Extreme Sleep Durations	Stroke risk	Not included in international statements.	Depicted a U-shaped relationship between extreme sleep durations (both reduced and long) and stroke risk. Pre-stroke extreme sleep durations have been associated with worse post-stroke functional and psychological outcomes, particularly among females and younger patients.

BDZ (Benzodiazepine); BDZRs (Benzodiazepine related drugs); CBT-I (Cognitive Behavioral Therapy for Insomnia); CPAP (Continuous Positive Airway Pressure); CRSWDs (Circadian Rhythm Sleep–Wake Disorders); CSA (Central Sleep Apnea); CVDs (Cardio-Vascular Diseases); DORAs (Dual Orexin Receptor Antagonists); GLP-1 (Glucagon-like Peptide 1); IH (Idiopathic Hypersomnia); LVO (Large Vessel Occlusion); MACEs (Major Adverse Cardiovascular Events); MCA (Middle Cerebral Artery); NT1/2 (Narcolepsy Type 1 or 2); OHS (Obesity-Hypoventilation Syndrome); OSA (Obstructive Sleep Apnea); PLMs (Periodic Limb Movements); RBD (REM Behavior Disorder); RLS (Restless Legs Syndrome); TMS (Transcranial Magnetic Stimulation).

**Table 2:** Average alterations of CAP rate and each A subtype in different sleep disorders, according to Parrino et al., 2012 [182] and Misirocchi et al., 2024 [196].

	CAP Rate %	A1%	A2%	A3%
Chronic insomnia	+	+	+	+
Severe Obstructive sleep apnea	+	-	=	+
Periodic limb movement disorder	+	-	+	+
Bruxism	+	-	+	+
NREM sleep parasomnia	+	+	=	=
Sleep-related epilepsies	+	+	+	+
Narcolepsy	-	-	+	=
Neurodegenerative disorders	-	=	=	=
REM sleep parasomnia	-	-	=	=
Chronic use of hypnotics	-	-	-	=

“+”: Increased value; “=”: Unaltered value; “-”: Reduced value.

## Data Availability

No new data were created or analyzed in this study. Data sharing is not applicable to this article.

## Abbreviations

The following abbreviations are used in this manuscript:

∆HR	Pulse rate response to respiratory events
AASM	American Academy of Sleep Medicine
AHA	American Heart Association
AHI	Apnea Hypopnea Index
ASA	American Stroke Association
BDZ	Benzodiazepine
BP	Blood Pressure
CAP	Cyclic Alternating Pattern
CBT-I	Cognitive Behavioral Therapy for Insomnia
CHS	Central Hypoventilation Syndrome
CPAP	Continuous Positive Airway Pressure
CRSWDs	Circadian Rhythm Sleep–Wake Disorders
CSA	Central Sleep Apnea
CSB	Cheyne-Stokes Breathing
CVD(s)	Cardiovascular Disease(s)
EAN	European Academy of Neurology
ERS	European Respiratory Society
ESO	European Stroke Organization
ESRS	European Sleep Research Society
GAD-7	General Anxiety Disorder-7
GLP1	Glucagon-like Peptide 1
HB	Hypoxia Burden
HPA	Hypothalamic–Pituitary–Adrenal axis
HR	Hazard Ratio
ICSD-3rd-TR	International Classification of Sleep Disorders, 3rd version revised
IH	Idiopathic Hypersomnia
IMT	Intima-Media Thickness
IPD	Individual Participant Data
iRLS	Idiopathic Restless Legs Syndrome
ISI	Insomnia Severity Index
MACEs	Major Cardiovascular Events
NIHSS	National Healthcare Stroke Scale
NT1/2	Narcolepsy type 1/2
ODI	Oxygen Desaturation Index
OHS	Obesity Hypoventilation Syndrome
OR	Odds Ratio
OSA	Obstructive Sleep Apnea
PAP	Positive Airway Pressure
PHQ-9	Patient Health Questionnaire-9
PLM(s)	Periodic Limb Movements
PLMD	Periodic Limb Movements Disorder
PSQI	Pittsburgh Sleep Quality Index
PTA	Peripheral Artery Tonometry
PTT	Pulse Transit Time
PWA	Pulse Wave Amplitude
RBD	REM sleep Behavior Disorder
RCTs	Randomized Controlled Trials
RLS	Restless Legs Syndrome
RR	Risk Ratio
SA	Sleep Apnea
SBDs	Sleep-related Breathing Disorders
SDs	Sleep Disorders
SHD	Sleep-related Hypoxemia Disorder
SHE	Sleep Hypermotor Epilepsy
TE-CSA	Treatment-Emergent Central Sleep Apnea
